# A bibliometric study of publications by Indian ophthalmologists and vision researchers, 2001-06

**DOI:** 10.4103/0301-4738.64117

**Published:** 2010

**Authors:** R Kumaragurupari, Pamela C Sieving, Prajna Lalitha

**Affiliations:** Library and Information Center, Madurai, Tamil Nadu, India; 1 National Institutes of Health Library, National Institutes of Health, Bethesda, Maryland, USA; 2 Microbiology Department, Aravind Eye Care System, Madurai, Tamil Nadu, India

**Keywords:** Bibliometrics, India, ophthalmic research, ophthalmology, vision research

## Abstract

**Objective:**

The objective was to conduct a bibliometric analysis of Indian ophthalmic papers published from 2001 to 2006 in the peer-reviewed journals, to assess productivity, trends in journal choice, publication types, research funding, and collaborative research.

**Materials and Methods:**

We searched PubMed for articles indicating both vision-related content and author affiliation with an Indian research center. We identified research collaborations and funding from indexing for research support, and classified articles as reporting basic science, clinical science, or clinically descriptive research. Impact factors were determined from Journal Citation Reports for 2006.

**Results:**

The total number of published articles that were retrieved for the years 2001 to 2006 was 2163. During the six-year period studied, the annual output of research articles has nearly doubled, from 284 in 2001 to 460 in 2006. Two-thirds of these were published in international journals; 41% in vision-related journals with 2006 impact factors; and 3% in impact factor journals which were not vision-related. Fifty percent of the publications came from nine major eye hospitals. Clinical science articles were most frequently published whereas basic science the least. Publications resulting from international collaborations increased from 3% in 2001 to 8% in 2006. The focus of the journal with the highest number of publications corresponds to the most common cause of bilateral blindness in India, cataract.

**Conclusion:**

This bibliometric study of publications of research from India in the field of ophthalmic and vision research shows that research productivity, as measured in both the number of publications in peer-reviewed journals and qualitative measures of those journals, has increased during the period of this study.

Scientific publications serve an important role in the scientific process, providing a link between the production of knowledge and its use.[1] The study of scientific publications in a particular field, based on international bibliographic data, is one of the most widely used methods to measure scientific achievement. It is well documented that the European Union and the United States are the leading powers in biomedical investigation and publications.[2] A systematic assessment of ophthalmology and vision-related research publication from India is not available. Without objective information about current research output it is difficult to plan for necessary improvements in infrastructure related to the understanding, treatment and prevention of eye diseases. Thus, it is important to accurately assess global and regional productivity of ongoing research in ophthalmology and vision. The primary objective of this study was to analyze the scientific literature generated by ophthalmologists, optometrists and vision researchers in Indian eye hospitals, academic and research institutions that appeared from January 2001 to December 2006 in peer-reviewed journals indexed by Medline. Other objectives were: to identify trends in ophthalmic research publications in India; to compare the contributions made by Indian ophthalmologists and vision researchers in Indian eye institutions; to examine the journals in which these articles were published by their impact factors; to classify the distribution of articles published, as basic science, clinical, or clinically descriptive and to identify the collaborative research patterns among Indian ophthalmologists and vision researchers.

## Materials and Methods

This study examined scientific publications generated by ophthalmologists, optometrists and researchers working in vision science in India through a systematic search of Medline using the PubMed interface. Throughout this paper, the terms 'ophthalmology' and 'ophthalmic' will be used inclusively to describe ophthalmology, optometry, and related basic science work concerning vision.

Records were retrieved using the search terms and strategy described in the Appendix.

A combination of natural language and MeSH (MEdical Subject Headings) terms to retrieve a comprehensive set of articles in the field of ophthalmology was developed. Retrieval was restricted to publications for which the first author was associated with an Indian institution using the "affiliation" field of the database.

The period of analysis was restricted to publication from January 2001 to December 2006 by using the "limit" function. The analysis was further limited to documents that were judged to be original ophthalmic contributions to scientific knowledge. Lecture notes, letters to the editor, brief communication and discussions were excluded from this analysis.

The data obtained from each citation and recorded in a spreadsheet included date of publication, author affiliation, publication type, journal title, and type of article. It was necessary to manually identify and exclude citations not related to ophthalmology and vision. In order to determine how many of these articles were published in journals with impact factors, the data was stratified according to the 2006 impact factors from Journal Citation Reports. All articles included were examined for classification into one of three categories: "basic science" (investigative) if experiments were performed on animals or *in vitro*, "clinical science" (investigative) if experiments were done involving humans, or "clinically descriptive" for case presentations and articles that reviewed diseases. These categories of data were identified using 'Animals' and 'Humans' in the "limit" options in the database; the "publication type" limit function was used for retrieving clinically descriptive publications. Another important analysis, which was identifying collaborative research between Indian researchers and investigators from other countries, was done by using "research support" from the US National Institute of Health, ("Research Support, N.I.H., Extramural" or "Research Support, N.I.H., Intramural").

## Results

The total number of articles identified from the Medline database between 2001 and 2006 was 2163, as shown in Table 1. The number of publications by Indian authors in journals indexed in Medline increased steadily during the early years of this decade; over the six-year period studied, the annual output of research articles has nearly doubled, from 284 in 2001 to 460 in 2006. The total number of articles that were published in 2001 was 13% whereas in 2006 it was 21%. Of these 2163 articles, 1448 (67%) were published in journals published outside India and 713 (33%) were published in Indian (national) journals.

**Table 1:** Publications by Indian ophthalmologists and vision scientists, 2001-06

Year	Publications
2001	284
2002	298
2003	347
2004	354
2005	420
2006	460
Total	2163

**Figure 1:**
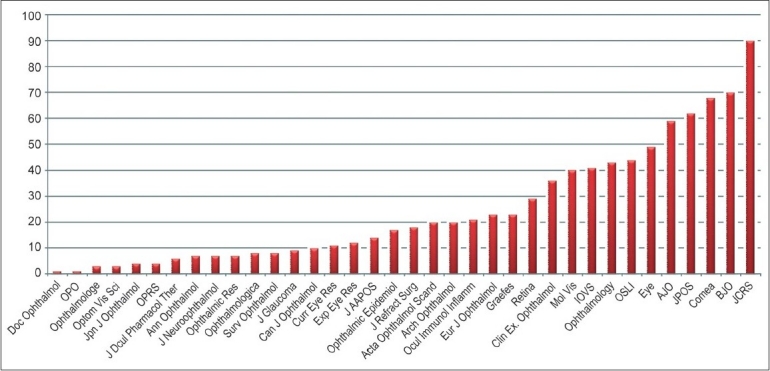
Distribution of articles by Indian authors in journal with 2006 impact factor OPO-Ophthalmic Physiol Opt; OPRS-Ophthal Plast Reconstr Surg; Graefes-Graefes Arch Clin Exp Ophthalmol; IOVS-Invest Ophthalmol Vis Sci; OSLI-Ophthalmic Surg Lasers Imaging; AJO-Am J Ophthalmol; JPOS-J Pediatr Ophthalmol Strabismus; BJO - Br J Ophthalmol; JCRS-J Cataract Refract Surg

The number of published articles was considered as an index of quantity of research productivity. Fig. 1 illustrates the distribution of articles by Indian authors in journals with 2006 impact factors, a measure of quality. According to the 2006 Ophthalmology journal impact factor, 41% of the articles (888/2163) were published in impact factor-ranked journals.

**Figure 2:**
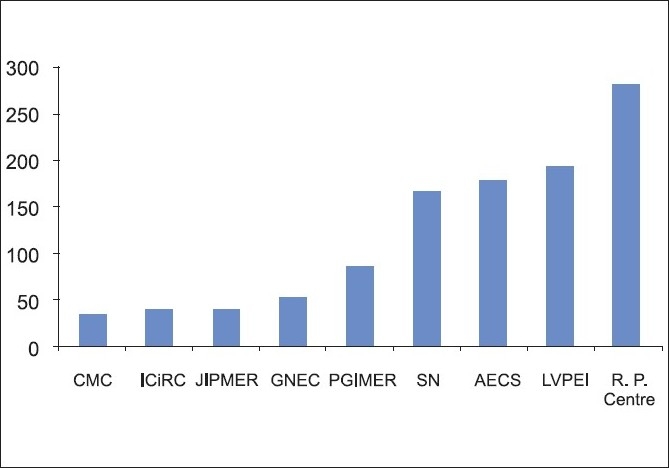
Research output of individual institutions CMC - Christian Medical College; ICIRC - Iladevi Cataract & IOL Research Centre; JIPMER - Jawaharlal Institute of Postgraduate Medical Education and Research; GNEC - Guru Nanak Eye Center; PGIMER - Postgraduate Institute of Medical Education & Research; SN - Sankara Nethralaya; AECS - Aravind Eye Care System; LVPEI - L. V. Prasad Eye Institute; R.P Center-R. P. Centre of Ophthalmic Sciences

Table 2 shows that a significant number of articles were also published in international non-ophthalmic journals with significant impact factors, including such titles as Human Genetics, Journal of Medical Genetics, Medical Science Monitor, Journal of Chemical Physics, and Anaesthesia and Intensive Care. Among the Indian journals, 42% (302/715) of articles were published in an ophthalmic journal, namely Indian Journal of Ophthalmology and 58% (413/715) were published in non-ophthalmic journals.Fig. 2 shows the number of articles published by the major eye institutes in India and the majority of the publications have been contributed by these institutes in 2001 to 2006 .

**Table 2:** Distribution of publications in selected non-vision journals with impact factors

Human Genetics	22
Journal of Chemical Physics	18
Anaesthesia and Intensive Care	9
Mycoses	7
Postgraduate Medical Journal	7
Medical Science Monitor	4
Journal of Medical Genetics	3

**Figure 3:**
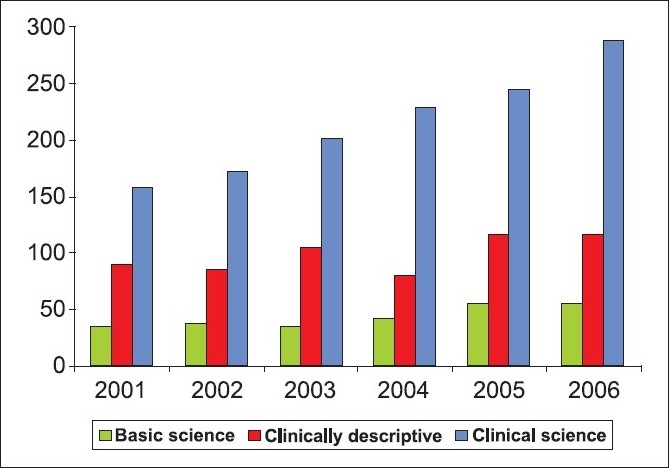
Classification of research publications

Using features of PubMed as described above, articles were classified as basic science, clinical science or clinically descriptive; the results are shown in Fig. 3 and Table 3. Overall results for the period under study showed that clinical science articles were consistently the largest percentage among the total articles published (60%); clinically descriptive articles were the second most prevalent (27%), and basic science comprised the smallest category (12%). In the year 2001, the number of articles in each of these categories was clinical science 159 (55%), clinically descriptive 90 (32%), and basic science 35 (12%), for a total of 284. In 2006, of the 460 total, basic science articles totaled 56 (12%), clinically descriptive articles increased to 115 (25%) and there were 289 (63%) clinical science articles.

**Table 3:** Classification of publications

Year	Clinically Descriptive	Basic Science	Clinical Science	Total	%
2001	90 (32)	35 (12)	159 (56)	284	13
2002	86 (29)	38 (13)	174 (58)	298	14
2003	105 (30)	38 (11)	204 (59)	347	16
2004	81 (23)	43 (12)	230 (65)	354	16
2005	117 (28)	57 (13)	246 (59)	420	19
2006	115 (25)	56 (12)	289 (63)	460	21
Total	594 /2163 (27)	267/2163 (12)	1302/2163 (60)	2163	100

Figures in parentheses are percentage

Table 4 highlights the fact that outside support for research in Indian institutions, as measured by publications, has increased steadily from 2001 to 2006. The annual number of papers resulting from collaborative research with supported researchers outside India increased from 69 to 175 over the period of six years, an increase from 3% to 8% of Medline-indexed papers by Indian researchers. This documents the increase in collaborative research between Indian ophthalmic researchers and those in such countries as the United Kingdom, France, Germany, Japan, and the United States.

**Table 4:** Research-supported publications

Year	Non US	US	Total	%
2001	64	5	69	3
2002	88	3	91	4
2003	118	7	125	5
2004	126	6	132	6
2005	137	6	143	7
2006	165	10	175	8
Total	698 (32%)	37 (2%)	745	34

## Discussion

This study looked at research productivity of Indian ophthalmologists, optometrists and vision researchers. According to a bibliometric analysis of publications from 1998 to 2002 by Ohba[3] the top 10 countries in terms of total number of articles published in the ophthalmic literature were the United States, the United Kingdom, Japan, Germany, Canada, Australia, Italy, Netherlands, Sweden, and France. India ranked 13^th^, with 1.3% of the total number of publications. In a study by Mandal it was found that researchers in the developing world contributed only 5.47% of the literature, compared to 92% from the developed world.[4]

Guerin *et al*.,[5] analyzed national productivity in five high-ranked ophthalmology journals for the 2002-06 publication years, correlating output with population demographics and research expenditures. They identified a 29% overall increase in productivity; for India, the number of publications in these five journals increased 123%, after Canada (143%) and China (131%). These papers provide an insight into the comparative position of Indian vision research productivity, but none examined the comprehensive publication records for a time period as recent as our study. Guerin provides intriguing correlations with economic support for research: The United States, which ranked first in ophthalmic research productivity, contributed 41% of the papers identified and devoted 2.68% of gross domestic product (GDP) to research and development, whereas India contributed only 0.61% of GDP, ranking ninth.

Analysis of the data in our study indicates that ophthalmic publications in India have shown a linear increase in year-by-year publications, with the total number doubling between 2001 and 2006. The subspecialty-related content of ophthalmic publications from India corresponds to the significance of the particular eye condition under study. We identified a correlation between the significance of cataracts as a cause of blindness in India and their significance in the research productivity of the country. Murthy *et al*., summarized the results of several population-based studies which found that in India, cataract is responsible for 50–80% of the bilaterally blind in the country.[6] These studies, funded by several agencies, including the government of India, the World Bank, and the Indian Council of Medical Research, were published in journals outside India, including those with the highest impact factors in ophthalmology. It is not surprising then that the highest number of articles by Indian vision researchers and ophthalmologists in a single journal were published in a journal devoted to cataract surgery and related research, The Journal of Cataract and Refractive Surgery.

Of the 2163 articles examined in our study, only 41% of the articles were published in journals with current impact factors. Liesegang *et al*., present concerns by journal editors about the source of funding for clinical studies. While research is expensive, and researchers may seek any available source of funding, editors are increasingly wary of commercial interests tainting the results of some research.[7] The low rate of non-commercial sponsorship of ophthalmic research in India, limiting the availability of funds to support this work, may be reflected in the relatively small percentage of articles published in the top 10 ranked journals (3%).

National journals should be encouraged and supported for publishing the work of Indian authors.[8,9] At the same time, publication in high impact factor journals is necessary for the advancement of Indian medical and scientific knowledge. In addition, for high impact factor journals to truly represent the best international content, their editors may need to actively seek international content, and provide dedicated space for publication from different regions, accepting only the best from these regions.

Many factors affect the ability of researchers in India to conduct high-quality research; among them is limited access to information resources. According to a survey by Murthy *et al*.,[10] subscriptions to international journals were inadequate in most of the institutions in India; their research in 2002-03 showed that only 53 of 128 surveyed ophthalmic institutions in India subscribed to more than two journals. The barriers to education, clinical care, research and vision care delivery created by lack of access to the ophthalmic literature may be partly overcome by new programs of free access to biomedical journals under a growing number of voluntary and mandated programs. If Indian researchers are careful in their choice of journals in which to publish their work, free access programs may also allow wider dissemination of the clinical and research data of Indian authors; this in turn may lead to increased citation of their work, and higher impact factors for the journals in which they publish. The Indian Journal of Ophthalmology is among the leaders in this movement, offering free access to the content of the journal since 1972.

Our data indicates that financial support by funding agencies for all three types of articles increased from 2001 to 2006. Clinical science and clinically descriptive articles had greater percentage increase in support than basic science articles; however, basic science articles consistently had the greatest overall percentage of citations of support, with approximately 80% of basic science articles acknowledging a funding source. According to Thomas, most ophthalmology training programs in India do not have a research component during the residency curriculum.[11] He identified some of the constraints which limit the capacity for meaningful research, including lack of research training, inadequate access to basic science facilities, inability to collaborate as full partners with non-medical scientists, lack of financial resources, and inadequate medical record standards.

A baseline report documenting programmatic and infrastructure resources and challenges as of 2005 is available.[12] This report highlights the strengths of the most productive programs, including those identified in our study, as well as areas for continued development which can be expected to result in further advances in Indian vision research.

International collaborative research supports many scientists and clinicians from India and has a growing record of biomedical accomplishment.[13] Many Indian researchers now have experience in collaborating with researchers in the US and other Western countries. They bring their own expertise; for instance, many have extensive experience in rural outreach, an important asset considering the need for comprehensive epidemiologic data and populations with both common and rare genotypes. Western scientists stand to gain enormously from access to the patients and expertise of their Indian collaborators.[14] As in Western societies, clinical and basic science research is increasingly seen as being an integral component of a well-balanced portfolio in many healthcare institutions in India. This is reflected by the increasing number of high-quality publications in leading eye journals. Areas in which Indian researchers have special expertise include genetics, basic biology of the eye, and informatics; these attract research collaborators.

This bibliometric study of publications in the field of ophthalmic and vision research in India shows that research productivity, as measured in both the number of publications in peer-reviewed journals and qualitative measures of those journals, has increased during the period of this study. The results of this study are an indicator of the productivity of Indian ophthalmologists and vision researchers. This analysis will be helpful to find out the obstacles of research productivity, which would help to develop research capacity and lead to more number of publications.

## APPENDIX

**Table:** SEARCH STRATEGY

Number	Search Terms	
1	Eye OR eye injury* OR eye manifestations OR eye diseases OR eye burns OR eye banks OR eye proteins OR eye foreign bodies OR ocular physiology OR ophthalmic assistants OR optometry OR lenses OR tears OR contact lens solutions OR eye movement OR eye measurements OR ophthalmi* OR ophthalmolog* OR ocular* OR optometr* OR retina* OR retini* OR macula* OR maculo* OR kerati* OR keratoca* OR keratoco* OR keratoe* OR keratok* OR keratop* OR glaucoma OR miotic* OR mydria* OR pupil* OR strabism* OR amblyop* OR saccad* OR bruch's OR retina OR uvea* OR low vision OR oculoplasty OR ocular microbiology OR blindness OR visual impairment OR neuro-ophthalmology OR orbit*	
2.	India OR Andhra Pradesh OR Hyderabad OR Secunderabad OR Tirupati OR Vijaywada OR Vishakhapatnam OR Warrangal OR Arunachal Pradesh OR Itanagar OR Assam Dispur OR Guwahati OR Tezpur OR Bihar OR Gaya OR Nalanda OR Patna Rajgir OR Vaishali OR Chhattisgarh OR Raipur OR Delhi OR Goa OR Mapusa OR Margao OR Old Goa OR Panaji OR Ponda OR Vasco Da Gama OR Gujarat OR Ahmedabad OR Gandhinagar OR Surat OR Haryana OR Chandigarh OR Faridabad Or Gurgaon OR Himachal Pradesh OR Chamba OR Kangra OR Kullu Manali OR Shimla OR Jammu and Kashmir OR Gulmarg Ladakh OR Jammu* OR Leh OR Srinagar OR Jharkhand Dhanbad OR Jamshedpur OR Ranchi OR Karnataka OR Bangalore Hampi Hassan OR Mangalore OR Mysore OR Udupi OR Kerala OR Alleppey Cochin OR Kovalam OR Kozhikode OR Munnar OR Quilon OR Trivandrum OR Lakshadweep OR Madhya Pradesh Aurangabad OR Mumbai OR Nagpur OR Pune OR Manipur Bhopal OR Indore Gwalior OR Orchha Khajuraho OR Maharashtra Kohima OR Orissa OR Bhubaneswar OR Cuttack OR Konark Or Puri OR Pondicherry OR Punjab OR Amritsar OR Chandigarh OR Ludhiana OR Patiala OR Rajasthan OR Ajmer OR Alwar OR Bikaner OR Bundi OR Jaipur Jaisalmer OR Jodhpur OR Shekhawati OR Udaipur OR Sikkim OR Gangtok OR Tamil Nadu OR Chennai OR Coimbatore OR Kanyakumari OR Kodaikanal OR Madurai OR Ooty OR Rameshwaram OR Thanjavur OR Trichy OR Tripura OR Agartala OR Uttaranchal OR Dehradun OR Haridwar Nainital OR Rishikesh OR Uttar Pradesh OR Agra OR Allahabad OR Lucknow OR Varanasi OR West Bengal OR Burdwan OR Darjeeling OR Durgapur OR Kolkata OR Murshidabad	
3	#1 AND #2	
4	Limits	Published Date: 2001 to 2006
